# The Arg/N-degron pathway mediates the secretion of apoptotic exosomes under oxidative stress in cancer cell

**DOI:** 10.1016/j.isci.2025.112637

**Published:** 2025-05-10

**Authors:** Su Bin Kim, Ji Su Lee, Chan Hoon Jung, Eun Hye Cho, Ho Seok Seo, Gee Eun Lee, Hye Yeon Kim, Su Jin Lee, Min Ju Lee, Hans Jin-young Oh, Ah Jung Heo, Do Hyun Han, Yong Tae Kwon, Chang Hoon Ji

**Affiliations:** 1 Cellular Degradation Biology Center and Department of Biomedical Sciences, College of Medicine, Seoul National University, Seoul 03080, Korea; 2 Department of Transdisciplinary Medicine, Seoul National University Hospital, 71 Daehakro, Seoul, Republic of Korea; 3 Structural Genomics Consortium, UNC Eshelman School of Pharmacy, University of North Carolina at Chapel Hill, Chapel Hill, NC 27599, USA; 4 Goethe University Frankfurt, Medical Faculty, Institute of Biochemistry II, Theodor-Stern-Kai 7, 60590 Frankfurt am Main, Germany; 5 AUTOTAC Bio Inc., 225 Gasan digital 1-ro, Seoul, Republic of Korea; 6 Ischemic/Hypoxic Disease Institute, College of Medicine, Seoul National University, Seoul 03080, Korea; 7 SNU Dementia Research Center, College of Medicine, Seoul National University, Seoul 03080, Republic of Korea

**Keywords:** Cell biology, Cancer

## Abstract

Exosomes from cancer cells are versatile mediators of cell-to-cell communication, whose cargoes are dynamically loaded in response to various stress conditions. In this study, we demonstrate that under oxidative stress, cancer cells secrete exosomes that induce apoptosis in neighboring cells via the Arg/N-degron pathway. In this mechanism, Rab interacting lysosomal protein (RILP) is cleaved at Asp75 in response to oxidative stress which requires ATE1 R-transferase. The cleaved form of RILP recruits the ESCRT-II proteins VPS22 and VPS36 to endosomes from which the interluminal vesicles are invaginated generating exosomes. By using proteomics analyses, we also demonstrate that exosomes secreted from cancer cells upon oxidative stress are enriched apoptotic proteins including pro-apoptotic and anti-inflammatory cytokine ANXA1. These exosomes induce apoptosis of normal cancer cells transporting ANXA1 in an Arg/N-degron pathway-dependent manner. Our results show that the Arg/N-degron pathway modulates exosome-mediated apoptosis in cancer cells under oxidative stress underlying RILP-dependent secretion of ANXA1.

## Highlights

• NSCLC cells secreted apoptotic exosomes under oxidative stress
• The Arg/N-degron pathway mediates pro-apoptotic, anti-inflammatory exosome secretion
• ANXA1 is an ATE1-mediated determinant cytokine in apoptotic exosomes
• ATE1-dependent RILP-ESCRT-II machinery is required for exosome biogenesis

## Graphical abstract

**Figure:**
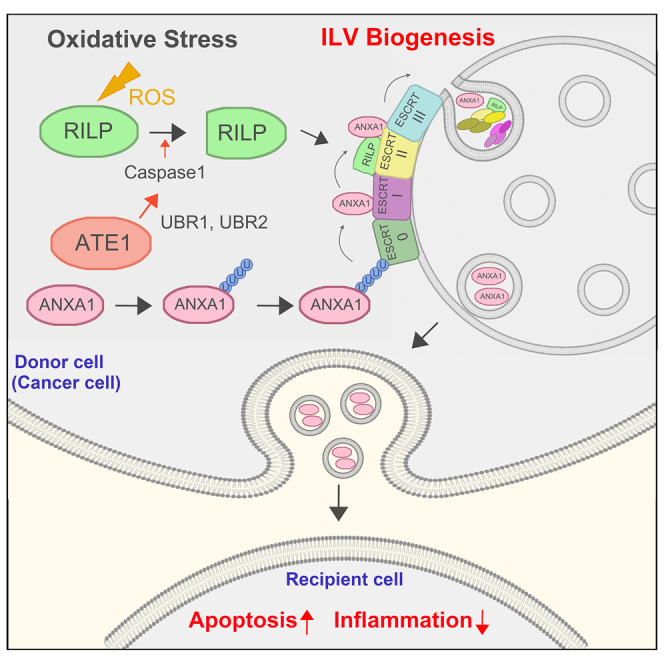

## Introduction

Exosomes are small extracellular vesicles secreted by various cells and play pivotal roles in intercellular communication by delivering distinct cargoes such as lipids, proteins, and nucleic acids to neighboring and distant cells, thereby propagating signaling cascades for biological processes such as development, immune response, and tissue regeneration.^1^^,^^2^^,^^3^ Under cellular stress conditions, the type and amount of cargo loaded into exosomes are dynamically shifted as a part of the cellular responses for both defense and signal propagation.^4^ Consequently, exosomes play an integral part in the pathogenesis and progression of a wide variety of diseases, delivering cargoes such as HMGB1 that can either promote or inhibit apoptosis, inflammation, proliferation, and differentiation in various cancer cells.^5^^,^^6^^,^^7^^,^^8^ However, the exact mechanisms and substrates underlying cargo loading in exosomes, especially in disease or stress conditions, have not yet been fully elucidated.

In the context of cancer cell-to-cell communication, cancer cell-derived exosomes can be a double-edged sword by transmitting both pro-survival/oncogenic and pro-apoptotic/tumor suppressor proteins depending on cellular stress conditions. Oxidative stress, which induces cell death and is implicated in the pathogenesis of a myriad of diseases, arises from an imbalance between the production and removal of reactive oxygen species (ROS).^9^^,^^10^^,^^11^^,^^12^^,^^13^ While oxidative stress can facilitate tumorigenesis in its early stages, most types of cancers and particularly lung cancer have been evolutionarily equipped to escape oxidative stress, as evidenced by oxidative stress responses that drive metastasis, invasion, angiogenesis, and vasculogenesis.^14^^,^^15^^,^^16^^,^^17^^,^^18^ Despite the importance of such oxidative stress responses and escape, the physiological relevance and mechanistic significance of oxidative stress-induced exosomes in cancer cells are yet unclear.

The N-degron pathway dictates the *in vivo* stability of substrate proteins based on the identity of their N-terminal amino acid residues (N-degrons) that are selectively recognized by E3 ligase or autophagy cargo receptor proteins called N-recognins.^19^ Within the Arg/N-degron pathway, type I and II N-degrons respectively include Arg, Lys, His (type 1), and Phe, Tyr, Trp, Leu, and Ile (type 2).^20^ Conjugation of L-Arg to N-terminal arginylation-permissive residues, namely aspartic acid, glutamic acid, and oxidized cysteine, is facilitated by the ATE1 R-transferase.^21^^,^^22^ ATE1-dependent Nt-arginylation has traditionally been associated with not only selective proteolysis of substrate proteins but also the maintenance of cellular proteostasis by removal of unwanted proteins, aggregates, organelles, and pathogens.^23^^,^^24^^,^^25^^,^^26^ While the functional role and significance of Nt-arginylation has been well-elucidated in terms of cellular degradation, its impact on other, non-degradative functions such as exocytosis or cellular signaling remains murky.^21^

Here, we characterized the role of the Arg/N-degron pathway in oxidative stress-induced cancer cell exosome biogenesis and secretion. Proteomic analysis revealed the effects of oxidative stress on exosomal cargo content, including upregulation of proteins associated with inflammation, apoptosis, and transport. The pro-apoptotic cytokine ANXA1 was secreted via oxidative stress-induced exosomes in an ATE1-dependent manner. Additionally, the ATE1 R-transferase was critical for the cleavage of RILP, thereby facilitating its recruitment to ESCRT-II proteins VPS22 and VPS36 for exosome biogenesis under oxidative stress. These results show that ATE1-dependent Nt-arginylation propagates cancer cell death under oxidative stress via secretion of pro-apoptotic exosomes.

## Results

### Exosomes secreted under oxidative stress induce cancer cell death

**Figure 1:**
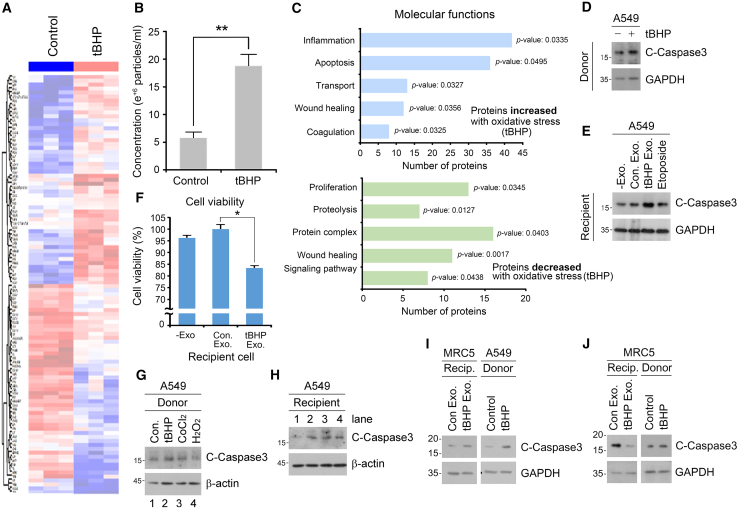
Characterization of oxidative stress-induced exosomes in cancer cells (A) Heatmap of all proteins identified in exosomes of A549 cells with tBHP (250 μM, 6 h) compared with control. The expression changes of proteins are shown on a relative scale, with depletion colored in blue and enrichment colored in red. (B) The concentration of exosome particles measured by nano-trafficking analysis. Exosomes were isolated from A549 cells in the presence or absence of tBHP (250 μM, 6 h). (C) Bar graph of Gene Ontology (GO) enrichment analysis. Enrichment of gene ontology terms within molecular pathways in increased and decreased exosomal proteins with oxidative stress. (D) Immunoblotting analysis of donor A549 cells with tBHP (500 μM, 6 h). (E) Immunoblotting analysis of A549 cells treated with oxidative stress-induced exosomes, as compared to those treated with the cancer cell death inducer, Etoposide (5 μM, 24 h). (F) Cell viability assay performed on the A549 cells in the presence or absence of exosome treatment extracted from control or tBHP (500 μM, 6 h) treated A549 cells. (G) Immunoblotting analysis of donor A549 cells with tBHP (250 μM, 6 h), CoCl_2_ (250 μM, 24 h), and H_2_O_2_ (100 μM, 6 h). (H) Immunoblotting analysis of A549 cells treated with oxidative stress-induced exosomes for 24 h. (I) Immunoblotting analysis of MRC5 treated with exosomes from A549 cells in the presence or absence of tBHP (250 μM, 6 h) for 24 h. (J) Immunoblotting analysis of MRC5 treated with exosomes from MRC5 cells in the presence or absence of tBHP (250 μM, 6 h) for 24 h. Error bars represent SEM (*n* = 3 replicates). ∗*p* < 0.05, ∗∗*p* < 0.01 using the paired t-test. See also Figures S1 and S2.

To test whether oxidative stress reprograms exosomal contents in cancer cells, particularly with respect to apoptosis and inflammation, exosomes were purified from non-small cell lung cancer (NSCLC) A549 cells in the presence or absence of the ROS generator *tert*-butyl hydroperoxide (tBHP) and analyzed via nano-trafficking analysis (NTA) (Figures S1A and S1B). LC-MS/MS data of the exosomal proteome identified ∼177 proteins whose intensity-based absolute quantification (iBAQ) were significantly up- (∼48%) or downregulated (∼52%) by oxidative stress (Figures 1A and S1C). Additionally, oxidative stress increased the number of exosome particles facilitating exosome secretion as determined by NTA (Figure 1B). In gene ontology analysis, the majority of upregulated proteins were associated with inflammation, apoptosis, transport, and wound healing (Figure 1C). On the other hand, the downregulated exosomal proteins were largely involved in proliferation, proteolysis, and protein-protein interaction/signaling (Figure 1C). Similar to tBHP treatment, the treatment of another oxidative stress inducer, cobalt chloride (CoCl_2_), also induced the secretion of proteins associated with apoptosis, transport, and wound healing (Figure S1D). In addition to these, proteins associated with localization, post-translational modification, proteolysis, and protein structures were secreted. In contrast, CoCl_2_ treatment downregulated exosomal proteins associated with transport, localization, proteolysis, wound healing, and signaling pathways, but not apoptosis (Figure S1D). These data suggest that oxidative stress alters the exosomal proteome of NSCLC cells by simultaneously promoting the secretion of pro-apoptotic, anti-tumorigenic proteins and withholding proteins required for constitutive cell function.

Next, we investigated the physiological role of these oxidative stress-induced exosomes via an *in vitro* exosome treatment assay. Briefly, exosomes were isolated via a polyethylene glycol-based solution from donor A549 cells treated with or without tBHP, and treated to recipient A549 cells. Immunoblotting analysis revealed that tBHP treatment induced apoptosis in donor A549 cells as determined by cleavage of the executioner caspase-3 (Figures 1D and S2A). Notably, the treatment of donor-derived exosomes to recipient A549 cells successfully cleavage of both caspase-3, to a similar degree as when recipient cells were treated with the DNA breakage stressor etoposide (Figures 1E and S2B). Additionally, flow cytometry of Annexin V/7-AAD confirmed that tBHP treatment-derived exosomes induced apoptosis in approximately 20% of recipient A549 cells, as opposed to 10% induced by basal exosomes (Figures S2C and S2D). Consistently, cell viability assays showed that recipient A549 cells treated with exosomes derived only from tBHP-treated donor A549 cells were significantly inhibited in cell proliferation by approximately 20% (Figure 1F). We intentionally isolated exosomes derived from donor cells not under excessive oxidative stress (Figure S2E), as programmed cell death is known to secrete other exosomes such as ApoEX containing the S1PR1/3 cytotoxic protein.^27^^,^^28^ In addition to tBHP, treatment of other oxidative stress inducers including CoCl_2_ and H_2_O_2_ also induced apoptosis in donor and recipient A549 cells (Figures 1G and 1H). To confirm whether the above phenomena were specific to cancer cells, we also performed the exosome treatment assays using normal lung fibroblast MRC5 cells as the donor. The treatment of MRC5-derived exosomes, in contrast to their NSCLC-derived counterparts (Figure 1I), to MRC5 recipient cells abolished oxidative stress-induced apoptosis (Figure 1J). To determine whether oxidative stress-induced exosomes from cancer cells were pro- or anti-inflammatory,^29^ we investigated the expression of pro-inflammatory cytokines in recipient cells. Interestingly, quantitative PCR analysis in A549 recipient cells following the same exosome treatment assay as above revealed a ∼60% and ∼80% downregulation of *IL-6* and *TNFα* mRNA levels (Figures S2F and S2G). Taken together, these results suggest that oxidative stress-induced exosomes derived from NSCLC cells are pro-apoptotic and anti-inflammatory.

### ATE1 regulates exosomal contents and cancer cell death under oxidative stress

**Figure 2:**
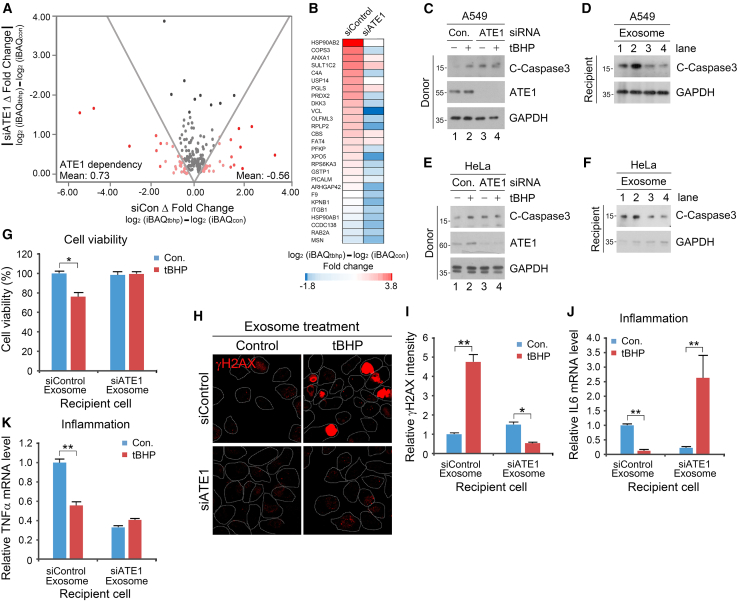
Requirement of ATE1 in pro-apoptotic and anti-inflammatory effects of oxidative stress-induced exosomes (A) A graph representing Δ fold change value of exosome components increased (right) and decreased (left) under oxidative stress. (B) Protein lists increased in an ATE1-dependent manner. (C) Immunoblotting analysis of A549 cells under *ATE1* knockdown and treated with tBHP (250 μM, 6 h). (D) Immunoblotting analysis of A549 cells treated with exosomes from C (24 h). (E) Immunoblotting analysis of HeLa cells under *ATE1* knockdown and treated with tBHP (250 μM, 6 h). (F) Immunoblotting analysis of HeLa cells treated with exosomes from E (24 h). (G) Cellular viability of recipient A549 cells with exosome originated from donor cells under *ATE1* knockdown and treated with tBHP (500 μM, 6 h). Results are presented as OD values (absorbance at 450 nm). (H) Immunostaining analysis of γH2AX in A549 cells with RNA interference of *ATE1* and treatment of tBHP (500 μM, 6 h). Scale bar, 10 μm. (I) Quantification of H (*n* = 150). (J) Relative mRNA level of *IL-6* in A549 cells treated with exosomes originated from cells under *ATE1* knockdown and tBHP (500 μM, 6 h) treatment compared with control. (K) Relative mRNA level of *TNFα* in A549 cells treated with exosomes originated from cells under *ATE1* knockdown and tBHP (500 μM, 6 h) treatment compared with control. Error bars represent SEM (*n* = 3 replicates). ∗*p* < 0.05, ∗∗*p* < 0.01 using the paired t-test. See also Figure S3.

We next determined whether Nt-arginylation as a post-translational modification plays what, if any, role in paracrine cell-to-cell communication of pro-apoptotic messengers in lung cancer cells. The iBAQ fold change ratio (Δfold change) for each exosomal cargo that was up- or downregulated upon tBHP-induced oxidative stress (Table S1) was calculated by subtracting their iBAQ fold change value (i.e., difference in iBAQ values prior and after tBHP treatment) in the presence or absence of *ATE1* interference (Figure S3A). The fold change ratios were deemed to be significant at greater than +0.3 or less than −0.3. Notably, for the exosome cargoes that were upregulated upon oxidative stress, the vast majority showed a decrease (mean value: −0.56) in their iBAQ fold change ratios upon *ATE1* knockdown (Figure 2A). Conversely, most of the exosome cargoes downregulated upon oxidative stress showed an increase (mean value: +0.73) in their fold change ratios upon *ATE1* knockdown (Figure 2A). Additionally, the ratio of oxidative stress-induced exosome cargoes with increased or decreased expression upon genetic depletion of ATE1 was essentially 1-to-1 (Figures S3A–S3C), indicating that Nt-arginylation is a determinant for proteins included and excluded in oxidative stress-induced NSCLC-derived exosomes. Among the 85 proteins upregulated post-oxidative stress, 27 proteins were filtered based on their ATE1-facilitated exosome loading (i.e., Δ fold change value < −0.3). These proteins included HSP90AB2, COPS3, ANXA1, SULT1C2, C4A, USP14, PGLS, PRDX2, DKK3, VCL, OLFML3, RPLP2, CBS, FAT4, PFKP, XPO5, RPS6KA3, GSTP1, PICALM, ARHGAP42, F9, KPNB1, ITGB1, HSP90AB1, CCDC138, RAB2A, and MSN (Figure 2B).

Oxidative stress-induced exosome treatment assays with donor cells in the presence or absence of *ATE1* siRNA revealed that the cleavage of caspase-3 within recipient A549 and HeLa cells was drastically abolished upon treatment of *ATE1*-interfered exosomes (Figures 2C–2F). Consistent with the above data, cell viability assays also showed that siRNA-mediated interference of *ATE1* rescued the oxidative stress-accelerated inhibition of cell proliferation, despite the pro-cell growth functions of the ATE1 R-transferase (Figure 2G). Immunostaining data consistently showed that the phosphorylated form of the histone variant H2AX (γH2AX), a marker of DNA double-strand breaks that occur during apoptosis, was upregulated (∼4.5-fold) in A549 cells treated with oxidative stress-induced exosomes (Figures 2H and 2I). However, genetic interference of *ATE1* completely abolished the above upregulation (Figures 2H and 2I). Additionally, genetic depletion of *ATE1* in donor cells generated pro-inflammatory exosomes (as opposed to the otherwise anti-inflammatory exosomes derived under normal ATE1 expression), as evidenced by the mRNA expression of *IL-6*, *TNFα,* and *IL-10* (Figures 2J, 2K, and S3D). These data indicate that ATE1 is necessary for the generation of pro-apoptotic and anti-inflammatory exosomes under oxidative stress.

### ANXA1 is an ATE1-mediated determinant cytokine of cancer cell death and inflammation under oxidative stress

**Figure 3:**
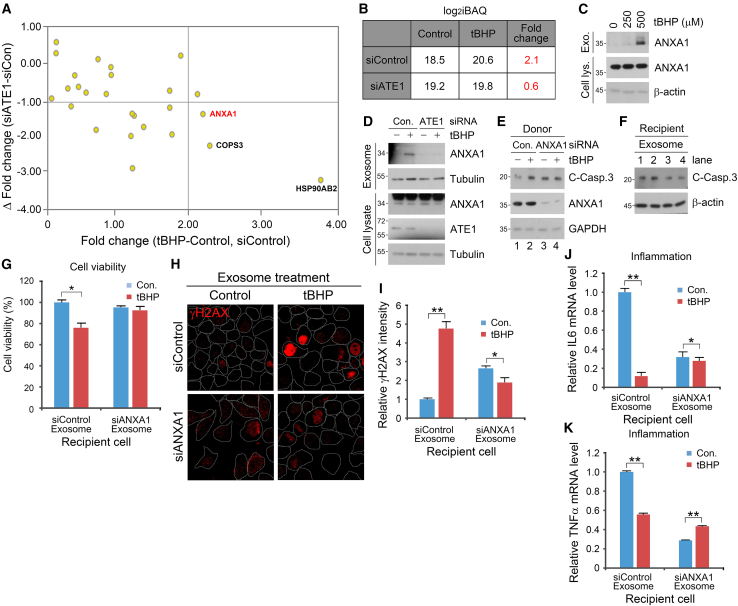
ANXA1 as an ATE1-mediated determinant cytokine inducing pro-apoptotic, anti-inflammatory, and pro-proliferative effects on recipient cells (A) A graph representing delta Δ fold change and Δ fold change of iBAQ. (B) A table regarding iBAQ level and fold change value of ANXA1 in secreted exosomes from A549 treated with tBHP (250 μM, 6 h) and RNA interference of *ATE1*. (C) Immunoblotting analysis of cell lysate and exosome fraction ANXA1 in A549 cells with 250 μM or 500 μM tBHP treatment (6 h). (D) Immunoblotting analysis of cell lysate and exosome fraction ANXA1 and Tubulin in A549 cells with RNA interference of *ATE1* and 250 μM tBHP treatment (6 h). (E) Immunoblotting analysis of A549 cells under *ANXA1* knockdown and treated with tBHP (250 μM, 6 h). (F) Immunoblotting analysis of A549 cells treated with exosomes from E (24 h). (G) Cellular viability of recipient A549 cells treated with exosomes originated from donor cells under *ANXA1* knockdown and treated with tBHP (500 μM, 6 h) (24h). Results are presented as OD values (absorbance at 450 nm). (H) Immunostaining analysis of γH2AX in A549 cells with RNA interference of *ANXA1* and treatment of tBHP (500 μM, 6 h). Scale bar, 10 μm. (I) Quantification of H (*n* = 150). (J) Relative mRNA level of *IL-6* in A549 cells treated with exosomes originated from cells under *ANXA1* knockdown and tBHP (500 μM, 6 h) treatment compared with control. (K) Relative mRNA level of *TNFα* in A549 cells treated with exosome originated from cells under *ANXA1* knockdown and tBHP (500 μM, 6 h) treatment compared with control. Error bars represent SEM (*n* = 3 replicates). ∗*p* < 0.05, ∗∗*p* < 0.01 using the paired t-test. See also Figure S4.

We next sought to identify the exosomal cargo responsible for inducing intercellular apoptosis and anti-inflammation upon oxidative stress. Among the 12 protein candidates that were targeted to oxidative stress-induced exosomes in an ATE1-dependent manner (i.e., |Δ fold change| > 0.3), HSP90AB2, COPS3, and ANXA1 were sorted based on their significant iBAQ fold change values (>2) and ratios (Δ < −1) (Figure 3A). Among these, Annexin 1 (ANXA1) is a well-known pro-apoptotic and anti-inflammatory cytokine.^30^^,^^31^ The iBAQ fold change values of ANXA1 under oxidative stress was reduced from 2.1-folds to 0.6-folds via genetic depletion of *ATE1* (Figure 3B), suggesting that the cytokine is loaded into exosomes almost solely via ATE1. Indeed, immunoblotting analysis corroborated that the oxidative stress-facilitated exosomal secretion of ANXA1 (Figure 3C) was decisively inhibited by siRNA-mediated knockdown of *ATE1* (Figure 3D). Interestingly, ANXA1 was secreted in an ATE1-dependent manner only during oxidative stress, but not other apoptotic stimuli as introduced by etoposide and doxorubicin treatment (Figure S4A).

Previous studies have reported ANXA1 as a double-edged cytokine responsible for inhibiting inflammation, promoting apoptosis or cell proliferation and facilitating cell migration/wound healing depending on the cancer cell type.^30^^,^^31^^,^^32^ We thus investigated whether ANXA1 is at least partially responsible for the pro-apoptotic and anti-inflammatory properties of oxidative stress-induced exosomes within NSCLC cells. Exosome treatment assays revealed that genetic interference of *ANXA1* in donor A549 cells mitigated the caspase-3 cleavage in counterpart recipient cells (Figures 3E and 3F). Cell viability assays also confirmed that ANXA1 regulates oxidative stress-mediated loss in cell proliferation (Figure 3G). Immunostaining analyses in A549 recipient cells treated with tBHP-induced exosomes also showed that genetic depletion of *ANXA1* abolished the increase in γ−H2AX signals, suggesting that ANXA1 is required for the apoptotic effect of oxidative stress-induced exosomes (Figures 3H and 3I). qPCR analysis of the pro-inflammatory cytokines IL-6 and TNFα in recipient A549 cells treated with oxidative stress-induced exosomes under siRNA-mediated interference of *ANXA1* revealed that ANXA1 is required for the anti-inflammatory properties of the donor-derived exosomes (Figures 3J and 3K). Moreover, qPCR analysis of the anti-inflammatory cytokine IL-10 in recipient cells under identical conditions as above showed that the increase in *IL-10* mRNA levels was drastically abolished upon siRNA-mediated knockdown of *ANXA1* in the donor cells (Figures S4B and S4C). To confirm that ANXA1 asserted these effects as a direct result of its secretion in an ATE1-dependent manner, we overexpressed ANXA1 in *ATE1-*interfered recipient cells. As suspected, genetic depletion of ATE1 in donor cells abolished the downregulation of IL-6 in recipient cells resulting from ANXA1 overexpression, despite the ∼4-fold increase in ANXA1 transcription in *ATE1-*interfered donor cells (Figures S4D and S4E). In addition to regulating programmed cell death and inflammation, ANXA1 is known to facilitate cell migration and wound healing.^30^^,^^31^ Wound healing assays showed that genetic interference of *ANXA1* in the donor cells under tBHP treatment generated exosomes which not only failed to activate but rather inhibited the wound healing process (Figures S4F and S4G). Taken together, our results show that the ANXA1 cytokine is responsible for the pro-apoptotic, pro-wound healing, and anti-inflammatory effects of oxidative stress-induced exosomes in cancer cells.

### ATE1-dependent interaction of VPS36 and VPS22 facilitates invagination of ANXA1^+^ ILVs

**Figure 4:**
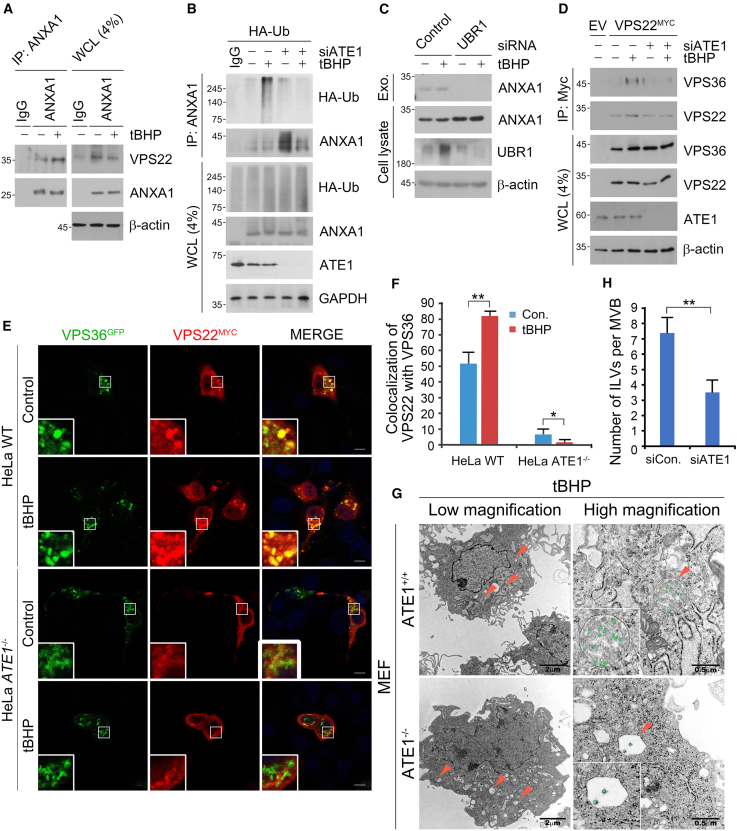
ATE1-dependent interaction of VPS36 and VPS22 facilitates invagination of ANXA1^+^ ILVs (A) Co-immunoprecipitation (Co-IP) analysis of the interaction between endogenous ANXA1 and VPS22 in A549 cells followed by tBHP (250 μM, 6 h) treatment. (B) Denaturation-IP analysis of the interaction between endogenous ANXA1 in A549 cells transfected with Ub^−HA^ under ATE1 knockdown followed by tBHP (250 μM, 6 h) treatment. (C) Immunoblotting analysis of cell lysate and exosome fraction ANXA1 in A549 cells with RNA interference of *UBR1* and *UBR2*, followed by tBHP treatment (250 μM, 6 h). (D) Co-IP analysis of the interaction between VPS36 and VPS22 in A549 cells transfected with VPS22^−MYC^ with RNA interference of *ATE1* followed by tBHP (250 μM, 6 h) treatment. (E) Co-localization immunostaining analysis of VPS22 with VPS36 in HeLa cells with RNA interference of *RILP* and treatment of tBHP (250 μM, 6 h). Scale bar, 10 μm. (F) Quantification of E (*n* = 50). (G) Transmission electron microscopy (TEM) image of WT and ATE1^−/−^ MEF cells with tBHP treatment (250 μM, 6 h). (H) Quantification of G (*n* = 30). Error bars represent SEM (*n* = 3 replicates). ∗*p* < 0.05, ∗∗*p* < 0.01 using the paired t-test. See also Figure S5.

As a necessary step in exosome maturation and secretion, the invagination of interluminal vesicles (ILVs) within multivesicular bodies (MVBs) is mainly mediated by endosome sorting complexes required for transport (ESCRT) proteins.^33^^,^^34^^,^^35^ Recognition of exosomal cargoes via ESCRT-0 simultaneously recruits ESCRT-II machinery components such as VPS22, VPS36, and VPS25 that initiate ILV invagination and eventual exosome formation.^36^ While distinct ESCRT proteins and their complexes have been characterized for the secretion of individual proteins in a myriad of cellular stress conditions, none have yet been identified for oxidative stress-induced exosome biogenesis, especially in cancer cells.^37^^,^^38^ Thus, we asked whether Nt-arginylation modulates the exosomal cargo loading machinery in cancer cells under oxidative stress, and what ESCRT regulators if any are involved. Co-immunoprecipitation analysis using ANXA1 as an Nt-arginylation-modulated model cytokine showed that tBHP-induced oxidative stress significantly promoted the interaction of ANXA1 with the ESCRT-II machinery component, VPS22 (Figure 4A).

Notably, denaturation immunoprecipitation assays showed that the oxidative stress-accelerated ubiquitination of ANXA1 with recombinant Ub^−HA^ was dependent on ATE1 (Figure 4B). To rule out the possibility that the above phenomena occurred in an ATE1-dependent, but Arg/N-degron pathway-independent manner, we isolated exosomes from A549 cells under genetic interference of the UBR E3 ligase/N-recognins *UBR1*, which selectively recognize the N-terminal arginine degron conjugated by the ATE1 R-transferase.^19^ Exosome treatment assays in donor A549 cells under genetic depletion of the *ATE1* or UBR E3 ligase/N-recognin *UBR1* confirmed that the ATE1 and UBR1 were required for the exosomal secretion of ANXA1 and ALIX, an auxiliary component of the ESCRT machinery first identified during viral budding, and also a marker for exosomal biogenesis (Figures 4C and S5A). Immunoblotting of exosomes also confirmed that genetic interference of the autophagic cargo receptor/N-recognin *p62*^39^ failed to abolish the secretion of ANXA1, but not that of ALIX (Figures S5A and S5B). Collectively, these data highlight the role of ATE1 and the Arg/N-degron pathway in ANXA1 ubiquitination, engagement with the ESCRT-II machinery, and exosomal secretion.

During exosome biogenesis, VPS22 interacts with VPS36 to form an ESCRT-II complex that marks the site of ILV invagination by recruiting ESCRT-III effectors.^36^ Thus, we sought out to determine whether the ANXA1-VPS22 complex recruits VPS36 during oxidative stress in an ATE1-dependent manner. Co-immunoprecipitation analysis showed that tBHP treatment promoted the formation of the VPS22-VPS36 complex, which was drastically inhibited upon siRNA-mediated knockdown of *ATE1* (Figure 4D). In addition, immunostaining analysis of CRISPR/Cas9-generated *ATE1*^−/−^ HeLa cells showed that the formation and co-localization of VPS36 and VPS22 puncta structures were absent (Figures 4E and 4F), in either basal or tBHP-induced oxidative stress. When the morphology of MVBs was analyzed using transmission electron microscopy (TEM), the formation of numerous ILVs (on average seven ILVs per MVB) was observed upon tBHP treatment in wild-type mouse embryonic fibroblasts (MEFs). *ATE1*^−/−^ MEFs, however, exhibited defective and relatively empty MVBs (Figures 4G and 4H). NTA analysis revealed that *ATE1* interference also reduced the overall number of exosome particles by ∼13% (Figure S5C). This raised the possibility that ATE1 mediates the formation of oxidative stress-induced exosomes since the VPS22-VPS36 complex is required for ILV invagination. Indeed, immunostaining analysis in HeLa cells showed that *ATE1* interference significantly abolished the co-localization between puncta structures of VPS22 and of the exosome marker CD81 (Figures S5D and S5E). In sum, these results highlight the necessity of the Arg/N-degron pathway and Nt-arginylation for ESCRT-II complex formation and subsequent exosome biogenesis via ILV invagination in cancer cells under oxidative stress.

### ATE1 is required for RILP cleavage and subsequent secretion of ANXA1 under oxidative stress

**Figure 5:**
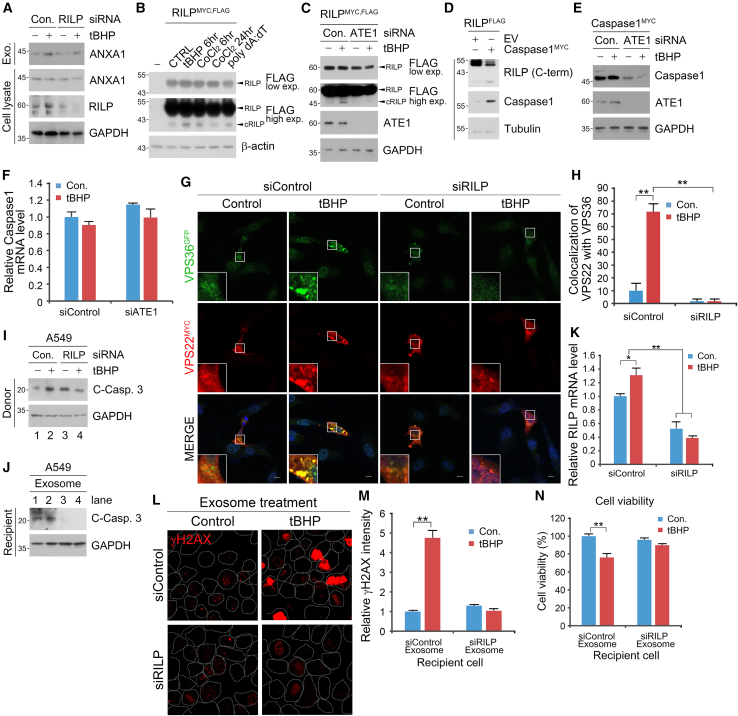
ATE1 is required for RILP cleavage and subsequent secretion of ANXA1 under oxidative stress (A) Immunoblotting analysis of cell lysate and exosome fraction ANXA1, RILP, and GAPDH in A549 cells with RNA interference of *RILP* and 250 μM tBHP treatment (6 h). (B) Immunoblotting analysis of A549 cells transfected with RILP^−MYC/FLAG^ with tBHP (250 μM, 6 h), CoCl_2_ (250 μM, 6 h), CoCl_2_ (250 μM, 24 h), or poly dA:dT (1 μg/μL, 24 h). (C) Immunoblotting analysis of A549 cells transfected with RILP^−MYC/FLAG^ with RNA interference of ATE1 and 250 μM tBHP treatment (6 h). (D) Immunoblotting analysis of cleaved C-term RILP in A549 cells transfected with RILP^−MYC/FLAG^, Caspase 1^−MYC^, or both. (E) Immunoblotting analysis of A549 cells under *RILP* knockdown and treated with tBHP (250 μM, 6 h). (F) Relative mRNA level of *Caspase-1* in A549 cells treated with exosome originated from cells under *ATE1* knockdown and tBHP (250 μM, 6 h) treatment compared with control. (G) Co-localization immunostaining analysis of VPS36 and VPS22 in WT and *ATE1*^−/−^ HeLa cells transfected with VPS36^−GFP^ and VPS22^−MYC^ with treatment of tBHP (250 μM, 6 h). Scale bar, 10 μm. (H) Quantification of G (*n* = 50). (I) Immunoblotting analysis of A549 cells under *RILP* knockdown and treated with tBHP (250 μM, 6 h). (J) Immunoblotting analysis of A549 cells treated with exosomes from I (24 h). (K) Relative mRNA level of *RILP* in A549 cells under *RILP* knockdown and tBHP (250 μM, 6 h) treatment compared with control. (L) Immunostaining analysis of γH2AX in A549 cells with RNA interference of *ANXA1* and treatment of tBHP (500 μM, 6 h). Scale bar, 10 μm. (M) Quantification of L (*n* = 150). (N) Cellular viability of recipient A549 cells with exosome originated from donor cells under *RILP* knockdown and treated with tBHP (500 μM, 6 h) (24 h). Results are presented as OD values (absorbance at 450 nm). Error bars represent SEM (*n* = 3 replicates). ∗*p* < 0.05, ∗∗*p* < 0.01 using the paired t-test. See also Figure S6.

Given that Nt-arginylation and the Arg/N-degron pathway modulates the *in vivo* proteolysis of substrate proteins, we further delved into how the ATE1 R-transferase mediates exosomal cargo loading and ILV invagination via the ESCRT-II complex. Previous studies have shown that Rab-interacting lysosomal protein (RILP), an effector of the endosome/exosome marker Rab7, recruits the ESCRT-II components VPS36 and VPS22 to the outer surface of secretory vesicle membranes.^40^ Specifically, while full-length RILP interacts with Rab7 for the delivery of endocytic vesicles or lysosomes toward the nucleus (minus end), cleaved RILP facilitates exocytosis via delivery of the vesicles toward the plasma membrane.^40^ Thus, we hypothesized that ATE1-facilitated Nt-arginylation is a determinant of RILP functionality for recruitment and delivery of the ESCRT-II complex components to the MVB membrane for the formation of pro-apoptotic exosomes under oxidative stress. Immunoblotting analysis of intracellular and exosomal fractions of A549 cells treated with tBHP revealed that RNA interference of *RILP* abolished the localization of ANXA1 into the oxidative-stress-induced exosomes (Figure 5A). Similarly, the oxidative stress-accelerated exosomal secretion of TSG101, an ESCRT-I component involved in ILV invagination from the MVB membrane, was also completely abolished upon RNA interference of *RILP* (Figure S6A). Interestingly, the total protein level of CD81, a transmembrane marker for exosomes, was not drastically affected in the same conditions, suggesting that RILP-mediated ILV invagination and exosome biogenesis was specific to oxidative stress (Figure S6A). Moreover, to investigate the precise mechanisms by which RILP mediates the exosomal trafficking of vesicles, we analyzed the interaction of RILP with ARL8B and Kinesin (KLC2) that are required for the anterograde movement of secretory vesicles.^41^^,^^42^ Co-immunoprecipitation and co-localization analysis revealed that oxidative stress facilitated the interaction of RILP with ARL8B and KLC2 into punctate structures (Figures S6B–S6D).

Next, we investigated whether RILP cleavage was mediated by ATE1. Immunoblotting analysis of A549 cells treated with acute or prolonged oxidative stress revealed that RILP was selectively cleaved only after acute treatment of tBHP or CoCl_2_, in direct contrast to the treatment of the DNA breakage stressor poly dA:dT (Figure 5B). Importantly, genetic depletion of *ATE1* inhibited the oxidative stress-driven cleavage of RILP (Figure 5C). However, in ATE1-deficient cells, overexpression of cleaved RILP facilitated the secretion of ANXA1 (Figure S6E). These data show that the ATE1-dependent RILP cleavage is the driving force behind oxidative stress-induced exosome formation in cancer cells. As RILP is known to be cleaved after Asp75 by caspase-1,^43^ we next assessed the relationship among ATE1-dependent Nt-arginylation, RILP, and caspase-1. Consistent with previous studies, ectopic expression of recombinant caspase-1 in A549 cells also induced RILP cleavage (Figure 5D). Notably, *ATE1* interference destabilized the inflammation-associated caspase-1 protein (Figure 5E) without affecting its transcriptional expression (Figure 5F). ATE1 facilitated the degradation of caspase-1, as determined by a reduction in caspase-1 half-life from six to 3 h (Figures S6F and S6G). These data indicate that ATE1 is required for the stability of caspase-1 and by extension caspase-mediated cleavage of RILP. Taken together, our results show that ATE1 modulates RILP cleavage and functionality via caspase-1 regulation.

To assess the effects of RILP on apoptosis and inflammation, immunocytochemical analysis of A549 cells showed that siRNA-mediated interference of *RILP* abolished the oxidative stress-induced co-localization of VPS36 and VPS22 punctate structures (Figures 5G and 5H). Consistently, *RILP* depletion generated defective exosomes under oxidative stress that do not induce caspase-3 cleavage (Figures 5I–5K), phosphorylation of H2AX (Figures 5L and 5M), and do not inhibit cell proliferation (Figure 5N) in recipient A549 cells. Notably, genetic depletion of *RILP* in donor cells failed to generate anti-inflammatory exosomes, as evidenced by the mRNA expression of *IL-6* and *IL-10* (Figures S6H and S6I). These data suggest that RILP is indeed a critical component for ATE1-mediated recruitment of ESCRT-II machinery for the formation of pro-apoptotic exosomes and their trafficking toward the plasma membrane in cancer cells under oxidative stress.

## Discussion

In this study, we showed that the ATE1 R-transferase and the Arg/N-degron pathway mediate pro-apoptotic and anti-inflammatory paracrine cell-to-cell communication among cancer cells under oxidative stress. Acute oxidative stress induced by ROS generated exosomes whose protein contents were positively associated with programmed cell death, wound healing, and anti-tumorigenesis. Oxidative stress-induced exosomes derived from cancer cells induced apoptosis and inhibited inflammation in an ATE1-dependent manner which was mediated by the exosomal secretion of the ANXA1 cytokine. Additionally, ATE1-dependent Nt-arginylation also regulated exosome biogenesis via the caspase1-facilitated truncation of the Rab7 effector RILP, whose resulting fragment formed a complex with the ESCRT proteins, VPS22 and VPS36. Thus, the Arg/N-degron pathway plays a bimodal role in accelerating cancer cell death under oxidative stress.

Many studies have elucidated the diverse molecular mechanisms, physiological relevance, and types of cell death for both normal and cancer cells.^44^^,^^45^ In extrinsic pathways, damage-associated molecular patterns (DAMPs) are released and recognized by specific receptors to convey the respective death signals. However, the exact mechanisms and regulators by which cancer cells attempt to escape the immune and oxidative stress response for their survival, and communicate such efforts to neighboring cancer cells are unclear. Our data show that the ANXA1 cytokine may play a vital and bimodal role in cancer cell death and survival under oxidative stress. Genetic depletion of *ANXA1* inhibited oxidative stress-induced cell death in donor A549 NSCLC cells, but instead generated pro-apoptotic and anti-inflammatory exosomes for neighboring cells. It will be interesting to investigate how ANXA1 functions as a double-edged sword for cancer cells, and whether this phenomenon is true for other cell death-inducing/inhibiting cytokines and signaling exosomal proteins in other types of cellular stress/cell death conditions.

While identification of specific cell death regulating cytokines, DAMPs, and other related proteins have been successful, the molecular principles and regulators behind the exosomal loading of these proteins are far from being understood. We provide here one possible scenario wherein the Arg/N-degron pathway controls the inflammation-associated caspase-1 expression or degradation for RILP cleavage, thereby recruiting the ESCRT-II machinery for simultaneous and energy-efficient cargo recognition/targeting in tandem with exosome biogenesis. Such identification may pave the way for further elucidation of how the Arg/N-degron pathway affects the cellular metabolism of caspase-1 and possibly other caspases. Additionally, pharmacological modulation of the Arg/N-degron pathway, including that of the yet-unidentified caspase-1 level regulator, may provide an alternative therapeutic avenue of targeting cancer cell death.

### Limitations of the study

This study shows that the Arg/N-degron pathway mediates the biogenesis of apoptotic exosomes in cancer cells. A critical remaining question is the identification of an Nt-arginylation-permissive substrate that regulates caspase-1 protein levels via either transcription or degradation, thereby affecting RILP cleavage and its downstream exosome formation in cancer cells. Moreover, this study focused on how ANXA1 underwent ubiquitination in an ATE1-and UBR N-recognin-dependent manner. Further studies should be performed to elucidate the recognition and delivery mechanisms for other exosomally secreted proteins under oxidative stress.

## Resource availability

### Lead contact

Further information and requests for resources and reagents should be directed to and will be fulfilled by the lead contact, Chang Hoon Ji (changhoon.ji@snu.ac.kr).

### Materials availability

This study did not generate new unique reagents.

### Data and code availability

• Original data from immunoblotting or immunocytochemistry assays are available at Mendeley Data under the following link (Mendeley data: https://data.mendeley.com/datasets/92mrpz7xx3/1). All software used are listed in the key resources table.
• The mass spectrometry proteomics data have been deposited to the ProteomeXchange Consortium via the PRIDE partner repository with the dataset identifier PXD048498 (Database: https://www.ebi.ac.uk/pride/archive/projects/PXD048498).
• Any additional information required to reanalzse the data reported in this paper is available from the lead contact upon request.

## Acknowledgments

We would like to thank the Korean Research Institute of Bioscience and Biotechnology (KRIBB) for their kind contribution of the wild-type and *ATE1*^−/−^ HeLa cells. This work was funded by the Basic Science Research Programs of the NRF funded by the Ministry of Science, ICT, and Future Planning (10.13039/501100003621MSIP) (NRF-2020R1A5A1019023 and NRF-2021R1A2B5B03002614 to Y.T.K.) and Korea Health Technology R&D Project through the Korea Health Industry Development Institute and Korea Dementia Research Center (KDRC) funded by the 10.13039/501100003621MSIP (RS-2024-00447844 to C.H.J.), and the 10.13039/501100003725National Research Foundation of Korea (NRF) funded by the Ministry of Education (RS-2023-00249464 to C.H.J.; RS-2024-00446110 to S.B.K.; and RS-2024-00461291 to C.H.J. and S.B.K.).

## Author contributions

Conceptualization and project administration: S.B.K., A.J.H., and C.H.J.; supervision: Y.T.K., D.H.H., and C.H.J.; Investigation: S.B.K., C.H.J., E.H.C., D.H.H., H.S.S., G.E.L., J.S.L., S.J.L., and M.J.L.; validation: S.B.K., J.S.L., H.Y.K., and H.J.O.; visualization: S.B.K., C.H.J., and H.S.S.; writing – original draft: S.B.K., C.H.J., E.H.C., H.S.S., J.S.L., and C.H.J.; writing – review and editing: S.B.K., C.H.J., E.H.C., H.S.S., G.E.L., J.S.L., S.J.L., M.J.L., H.Y.K., H.J.O., D.H.H., Y.T.K., and C.H.J.; funding acquisition: S.B.K., Y.T.K., and C.H.J. All authors contributed to the article and approved the submitted version.

## Declaration of interests

The authors declare that the research was conducted in the absence of any commercial or financial relationships that could be construed as a potential conflict of interest.

## STAR★Methods

### Key resources table

**Table:** 

REAGENT or RESOURCE	SOURCE	IDENTIFIER
**Antibodies**		

Mouse monoclonal Anti-FLAG_M2	SIGMA-ALDRICH	CAT#F1804; RRID: AB_262044
Rabbit polyclonal Anti-FLAG	SIGMA-ALDRICH	Cat#SAB4301135; RRID: AB_2811010
Rabbit polyclonal anti-Myc(71D10)	Cell signaling	Cat#2278S; RRID:AB_10693332
Rabbit polyclonal Anti-GAPDH	Bioworld	Cat#AP0066; RRID: AB_2797448
Mouse monoclonal Anti-beta-actin	SIGMA-ALDRICH	CAT#A1978; RRID: AB_476692
Mouse monoclonal Anti-C-Myc(9E10)	Santacruz	Cat#sc-40; RRID:AB_627268
Mouse monoclonal Anti-hATE1(H-12)	Santacruz	Cat#sc-271219; RRID: AB_10613800
Rabbit polyclonal Anti-RILP	Invitrogen	Cat#PA5-34357; RRID: AB_2551709
Rabbit polyclonal Anti-cleaved specific RILP	Invitrogen	Cat#PA5-107015; RRID: AB_2817731
Rabbit polyclonal Anti-VPS22 (SNF8)	Merck	Cat#SAB1410176; RRID: AB_3697206
Rabbit polyclonal Anti-VPS36	Invitrogen	Cat#PA5-58905; RRID: AB_2649462
Rabbit monoclonal Anti-CD81	SIGMA-ALDRICH	Cat#SAB3500454; RRID: AB_10640751
Rabbit polyclonal Anti-PAPR	Cell signaling	Cat#9542; RRID: AB_2160739
Rabbit polyclonal Anti-cleaved caspase3	Cell signaling	Cat#9662; RRID: AB_331439
Mouse monoclonal Anti-TSG101	Santacruz	Cat#sc-7964; RRID: AB_671392
Rabbit polyclonal Anti-ANXA1	Bethyl	Cat#A305-235A; RRID: AB_2631627
Mouse monoclonal Anti-p62	Abcam	Cat#:ab56416; RRID:AB_945626
Mouse monoclonal Anti-ALIX	Santacruz	Cat#sc-53540; RRID:AB_673819
Rabbit polyclonal Anti-H2A.X (Phospho S139)	Abcam	Cat#ab11174; RRID: AB_297813
Goat Anti-mouse IgG HRP	Invitrogen	Cat#31430; RRID: AB_228307
Goat Anti-rabbit IgG HRP	Invitrogen	Cat#31460; RRID: AB_228341
Alexa Fluor 555 goat anti-Rabbit IgG	Invitrogen	Cat#A21429; RRID: AB_2535850
Alexa Fluor 555 goat anti-Mouse IgG	Invitrogen	Cat#A21424; RRID: AB_141780
Alexa Fluor 488 goat anti-Rabbit IgG	Invitrogen	Cat#A11034;RRID:AB_2576217
Alexa Fluor 488 goat anti-Mouse IgG	Invitrogen	Cat#A11029; RRID:AB_138404
Normal mouse IgG	Santacruz	Cat#sc-2025; RRID:AB_737182
Normal rabbit IgG	Cell signaling	Cat#2729s; RRID: AB_1031062

**Chemicals, peptides, and recombinant proteins**		

tBHP (*tert-*butyl hydroperoxide)	SIGMA-ALDRICH	Cat#458139-25ML
Cocl_2_	SIGMA-ALDRICH	Cat#C8661-25G
Etoposide	SIGMA-ALDRICH	Cat#341205
Doxorubicin	SIGMA-ALDRICH	Cat#D1515
Poly dA:dT	Invivogen	Cat#tlrl-patn
DMEM	Life Technologies, Gibco®	Cat#11995-065
RPMI	Life Technologies, Gibco®	Cat#22400-089
Lipofectamine 2000 Transfection Reagent	Thermo Fisher scientific	Cat#11668019
Lipofectamine 3000 Transfection Reagent	Thermo Fisher scientific	Cat#13778150
SuperSignal™ West PICO PLUS	Thermo Fisher Scientific	Cat#34578
Opti-mem	Life Technologies, Gibco®	Cat#31984-070
4× Laemmli sample buffer	Biorad	Cat#1610747
Lipofectamine RNAiMAX Transfection Reagent	Thermo Fisher scientific	Cat#13778030

**Critical commercial assays**		

Exosome isolation Kit	Invitrogen	Cat#:447859
primescripTM 1st strand cDNA synthesis kit	Takara	Cat#6110A
2X Fast Q-PCR Master Mix (SYBR)	SMOBIO	Cat#TQ1210
D-Plus™ CCK cell viability assay kit	Dong-in Biotech	Cat#CCK-3000
Annexin V and 7-AAD apoptosis kit	Biotium	Cat#30060

**Deposited data**		

Mass spectrometry data	This paper: PRIDE	PXD048498

**Experimental models: Cell lines**		

A549	ATCC	Cat#CCL-185 RRID: CVCL_0023
HeLa	ATCC	Cat#CCL-2; RRID:CVCL_0030
HEK 293T	ATCC	Cat#CRL-11268; RRID;CVCL_1926
*P62*^*-/-*^ HeLa	KRIBB	
*ATE1*^*-/-*^ HeLa	KRIBB	

**Oligonucleotides**		

siRNA against Control sense	Bioneer	SN-1003
siRNA against ATE1 sense: ACCCA CCAUCUUUGUUUCCACCAAA	This paper	
siRNA against ATE1 anti-sense: UUUGG UGGAAACAAAGAUGGUGGGU	This paper	
siRNA against ANXA1 sense: UGACC GAUCUGAGGACUUU	This paper	
siRNA against ANXA1 anti-sense: AAAGU CCUCAGAUCGGUCA	This paper	
siRNA against ANXA1 sense: GAUCA AGGCCAAGAUGUUA=tt	This paper	
siRNA against ANXA1 anti-sense: UAACAU CUUGGCCUUGAUC=tt	This paper	
siRNA against p62 anti-sense: GUGAACUCCAGUCCCUACA	This paper	
siRNA against p62 anti-sense: UGUAGGGACUGGAGUUCAC	This paper	
siRNA against RILP anti-sense: GAUCAAGGCCAAGAUGUUA=tt	This paper	
siRNA against RILP anti-sense: UAACAUCUUGGCCUUGAUC=tt	This paper	
Primer IL-6 Forward: AGACAGCC ACTCACCTCTTCAG	This paper	
Primer IL-6 Reverse: TTCTGCCAGT GCCTCTTTGCTG	This paper	
Primer TNF-a Forward: GTCTCCTAC CAGACCAAG	This paper	
Primer TNF-a Reverse: CAAAGTAGAC CTGCCCAGACTC	This paper	
Primer IL-10 Forward Forward: TCTCCGAGATGCCTTCAGCAGA	This paper	
Primer IL-10 Reverse Reverse: TCAGACAAGGCTTGGCAACCCA	This paper	
Primer Caspase-1 Forward: TTACA GACAAGGGTGCTGAACAA	This paper	
Primer Caspase-1 Reverse: TGAGGAG CTGGAAAGGAAGAAAG	This paper	
Primer RILP Forward: GCAGCGGA AGAAGATCAAGGC	This paper	
Primer RILP Reverse: GACAAAGGT GTTCGTGGAGGG	This paper	
Primer ANXA1 Forward: AGCGTCAACA GATCAAAGCAGCAT	This paper	
Primer ANXA1 Reverse: AGACCCTGTT AATGTCTCTGATTT	This paper	

**Recombinant DNA**		

RILP-MYC,FLAG	Origene	Cat#RC206400
Cleaved-RILP-MYC,FLAG	This paper	
VPS22-MYC,FLAG	Origene	Cat#RC202137
VPS36-GFP	Origene	Cat#RG237476
Caspase1-MYC	Addgene	Cat#41552
ARL8B-V5	Addgene	Cat#67446
KLC-MCHERRY	Addgene	Cat#62747
ANXA1-MYC,FLAG	Origene	Cat#RC201569

**Software and algorithms**		

Zen blue edition	Zeiss	
Reichert Ultracut S Ultramicrotome	Leica Microsystems	
FEI Vitrobot Mark IV	Thermo Fisher Scientific	
BD FACSDiva™	BD Biosciences	
MaxQuant version 1.6.1.0	Max Planck Institute of Biochemistry	

**Other**		

Q-Exactive Plus	Thermo Fisher Scientific	
Ultimate 3000 RSLCnano system	Dionex	
BD FACS Canto II	BD biosciences	
JEM-1400 TEM	JEOL Ltd	

**Deposited data**		

	ProteomeXchange	PXD048498

### Experimental model and study participant details

#### Cell lines

Cell lines originated from human, A549, HeLa, and HEK 293T, were purchased from the American Type Culture Collection. Wild-type and *ATE1*^-/-^ and *p62*^*-/-*^ HeLa cells were purchased from Korea Research Institute of Bioscience and Biotechnology. All of the cells were authenticated using short tandem repeat (STR) profiling and routinely tested for mycoplasma contamination using PCR-based assays. A549 cells were cultured in Roswell Park Memorial Institute (22400-089; Life Technologies, Gibco®) supplemented with 10% FBS (16000044; Life Technologies, Gibco®) in a standard 37 °C, 5% CO_2_ incubator. HeLa and HEK 293T cells were cultured in Dulbecco’s Modified Eagle’s Medium (11995-065; Life Technologies, Gibco®) supplemented with 10% FBS in a standard 5% CO_2_ incubator.

### Method details

#### Cell transfection

To transfect plasmids into HEK293T and HeLa, lipofectamine 2000 (11668019; Invitrogen) was used. For A549, lipofectamine 3000 (L3000015; Invitrogen) was used. Each plasmid and lipofectamine were incubated in Opti-MEM media (31984-070; Life Technologies, Gibco®) before being treated in the cells.

#### Western blotting (WB)

Cells were harvested from plates using Phosphate-Buffered Saline (P2007-1; Biosesang) with 0.05% trypsin (25300; Life Technologies, Gibco®). The obtained cell pellets were lysed using an SDS-based sample buffer containing beta-mercaptoethanol (277.8 mM Tris-HCl, pH 6.8, 4.4% LDS, 44.4% (v/v) glycerol). Whole cell lysates were separated by SDS-PAGE and transferred onto polyvinylidene difluoride membranes. The membranes were blocked with 2% skim milk (SM2010; Georgiachem) in standard PBST (PBS and 0.1% Tween 20) wash buffer for 1 h, followed by incubation with primary antibodies in skim milk for 1 h, washing with PBST buffer, and incubation with secondary antibodies in PBST for 1 h. Immunoreactive proteins were visualized using SuperSignal™ West PICO PLUS (34578; Thermo Fisher Scientific) according to the manufacturer’s instructions and the emitted light was recorded on X-ray films.

#### Co-immunoprecipitation (Co-IP)

Cells were harvested and resuspended in 500 μl of lysis buffer (50 mM Tris-HCl pH 7.5, 150 mM NaCl, 0.5% Triton X-100, 1 mM EDTA, 1 mM PMSF, and protease inhibitor cocktail). The cells were incubated on a rotator for 30 min at 4°C and centrifuged at 13000 rpm for 10 min and debris was removed. The supernatants were pre-cleared by using IgG (normal Mouse IgG: sc-2025; Santa Cruz, normal Rabbit IgG: 2729s; Cell signaling,) and incubated with A/G agarose beads (Santa Cruz, sc-2003) for 3 h on the rotator at 4°C. Some portion of the supernatants were remained for western blotting (whole cell lysate). Then, the beads were centrifuged at 13000 rpm for 10 min and removed. Primary antibodies were injected into the supernatants and incubated on a rotator at 4°C overnight. Samples were added by beads and incubated on rotator at 4°C for 2 h 30 min. The beads were centrifuged at 5000 rpm for 2 min and washed with lysis buffer. These washing steps were repeated four times, and the proteins conjugated with beads were extracted in 2× Laemmli sample buffer (1610747; Bio-Rad) by heating for 10 min at 95°C. Proteins of interest were detected by immunoblotting.

#### Immunocytochemistry (ICC)

Cells were cultured on cover slips and fixed with 4% paraformaldehyde (pc2031-100-00; Biosesang) in PBS (pH 7.4) for 10 min at room temperature. After washing three times with PBS, the cells were permeabilized with 0.5% Triton X-100 (T9284; Sigma) for 25 min at room temperature. After three washes with PBS, the cells were incubated with blocking solution (2% BSA in PBS) for 1 h and then with primary antibodies overnight at 4°C. The next day, the cells were washed three times with PBS for 10 min each and then incubated with secondary antibodies for 35 min. The cells were washed three times with PBS for 10 min each, and after staining with DAPI (H-1500; Vectashield), the coverslips were mounted on slides.

#### RNA extraction and qRT-PCR analysis

The RNA from cells treated with drugs and exosomes was isolated using Trizol reagent (15596026; Invitrogen). Subsequently, cDNA was synthesized from 2 μg of extracted mRNA by using the Primescript™ 1st strand cDNA synthesis kit (6110A; Takara). The resulting synthesized cDNAs were then diluted to 1:4 using 40 μl of distilled water, and 4 μl of this diluted cDNA was utilized for quantitative RT-PCR. To assess the expression of specific genes, 2x Fast Q-PCR master mix with SYBR (TQ1210; SMOBIO) was employed.

#### Exosome purification

Conditioned media were harvested and centrifuged at 2000 xg for 30 min and the supernatants were transferred to new tubes. The samples were mixed with an Exosome isolation kit (4478359; Thermo Fisher) solution whose volume was half of the volume of the samples. Then the mixtures were incubated overnight and centrifuged at 10000 xg for 1 h. The purified exosomes were resuspended with PBS and added by 2× Laemmli sample buffer (1610747; Bio-Rad) with 10% beta-mercaptoethanol. Consequently, lysed samples were used for Immunoblotting.

#### Cell viability assay

5×10^4^ cells/well were seeded in a 96-well plate and incubated for 1 day. Cells were treated with exosomes derived from A549 cells that were treated with tBHP, siRNA transfection, or both. The cells were incubated with D-Plus™ CCK cell viability assay kit (CCK-3000; Dong-in Biotech) for 2 h in a 37°C incubator. The optical density (OD) value of samples was measured at 450 nm.

#### Flow cytometry

2×10^6^ cells/well were seeded in a 12-well plate and incubated for 1 day. Cells were treated with exosomes derived from A549 cells that were treated with tBHP. The cells were harvested and resuspended with 100 μl of 1× binding buffer. The cells were incubated with CF488A Annexin V and 7-AAD working solution for 40 min at room temperature. After added with 400 μl of 1× binding buffer, all the samples were analyzed using BD Canto II Flow Cytometry, which can detect FICT and PF-Cy7 channels.

#### Electron microscopy

WT and *ATE1*^*-/-*^ MEF cells were carefully harvested and centrifuged at 8000 rpm for 5 min. The pellets were resuspended in 2.5% glutaraldeyhyde in 0.1 M sodium cacodylate buffer (pH 7.4) (16537-20; Electron Microscopy Sciences) and incubated at 4°C overnight. Then the pellets were incubated with cacodylate buffer for 6 h and embedded in Epon resin. Then, samples were cut into 55-nm and stained with uranyl acetate and led citrate using the Reichert Ultracut S Ultramicrotome (Leica Microsystems) and FEI Vitrobot Mark IV (Thermo Scientific), respectively. The sections were observed using the JEM-1400 transmission electron microscope at the Seoul National University Hospital Biomedicine Research Institute.

#### Mass spectrometry

Exosomal protein sample preparation was performed using an optimized protocol that integrated filter-aided sample preparation and StageTip desalting, as detailed in previous studies.^46^ Briefly, denaturation buffer (2% SDS, 50 mM chloroacetamide, 10 mM Tris (2-carboxyethyl) phosphine hydrochloride in 0.1 M Tris-HCl pH 8.5) was added to the protein sample. For reduction and alkylation, the mixtures were heated at 95°C for 15 min. The protein samples were loaded onto a 30 kDa MWCO centrifugal filter (Amicon, Merck Millipore). Trypsin digestion was performed overnight at 37°C, with a protease-to-protein ratio (w/w) of 1:100. After acidifying with 10% trifluoroacetic acid (TFA), peptides were desalted using the StageTip method with Styrenedivinylbenzene-Reveres Phase Sulfonate.

LC-MS/MS analysis was conducted using a Q-Exactive Plus (Thermo Fisher Scientific) coupled with an Ultimate 3000 RSLCnano system (Dionex).^47^ Peptides were separated using a two-column system with a trap column (C18, 75 μm I.D x 2 cm length, 3 μm) and an analytic column (EASY-Spray C18, 75 μm I.D. × 50 cm length, 2 μm). The separation was achieved over a 120 min gradient ranging from 5% to 30% ACN at a flow rate of 300 nl/min. MS measurement was conducted in the positive ion mode. For data-dependent acquisition, a survey scan was conducted over the 350 to 1,800 m/z range, and the resolution was set at 70,000. The top-15 precursor ion was selected with an isolation window of 1.2 m/z. The MS/MS spectrum was acquired using a high collision dissociated-normalized collision energy of 30% and the resolution was set at 17,500.

MS raw files were processed using MaxQuant version 1.6.1.0 (Max Planck Institute of Biochemistry).^48^ MS/MS spectra were searched against the reviewed Human UniProt protein sequence database (December 2014, 88,657 entries). Searches were performed using a 6-ppm precursor ion tolerance for total protein level analysis. A 20-ppm tolerance was applied for the MS/MS ion. Carbamido-methylation was set as the fixed modification, and N-acetylation of protein and oxidation of methionine were set as variable modifications. The peptide and protein identification were conducted with a false discovery rate (FDR) set at 1%. The Intensity Based Absolute Quantification (iBAQ) algorithm was used as part of the MaxQuant platform for label-free quantification.^49^ The mass spectrometry proteomics data have been deposited to the ProteomeXchange Consortium via the PRIDE partner repository with the dataset identifier PXD048498 (https://www.ebi.ac.uk/pride/archive/projects/PXD048498).

### Quantification and statistical analysis

For quantification data of immunocytochemistry and quantitative real-time PCR analysis, each set of experiments was triplicated and performed three times. The data were shown as mean ± S.E.M. of three independent experiments. P-values were determined using a two-tailed student’s t-test (degree of freedom = n-1). Statistical significance was determined as values of p < 0.05 (∗∗p < 0.01; ∗p< 0.05). Statistical analysis of the MS data was performed utilizing Perseus software (version 1.6.15.0).^50^ For identification of differentially expressed proteins, iBAQ intensities were log2-transformed. After filtering out proteins with at least 2/3 valid values in each group, imputation missing values were performed using a normal distribution (width = 0.3, down-shift = 1.8). Two-sample t-tests were employed, and a significance criterion of p-value < 0.05 was applied for each group comparison.

## Supplemental information

Document S1. Figures S1–S6 and Table S2

Table S1. List of proteins identified by mass analysis LC-MS/MS analysis of exosomes isolated from cells subjected to RNA interference of ATE1 and treated with tBHP (250 μM, 6 h), compared to control cells. Fold changes and P-values were calculated by comparing control cells with tBHP-treated cells. ATE1 dependency was determined using the method described in Figure S3A.
