# Supplementary material for: The Arg/N-degron pathway mediates the secretion of apoptotic exosomes under oxidative stress in cancer cell
Source: iScience. 2025 May 10;28(6):112637. doi: 10.1016/j.isci.2025.112637 (PMC12167491; doi:10.1016/j.isci.2025.112637)
Supplement: Document S1. Figures S1–S6 and Table S2 [file mmc1.pdf]

iScience, Volume *28*

Supplemental information

The Arg/N-degron pathway mediates the secretion of apoptotic exosomes under oxidative stress in cancer cell

Su Bin Kim, Ji Su Lee, Chan Hoon Jung, Eun Hye Cho, Ho Seok Seo, Gee Eun Lee, Hye Yeon Kim, Su Jin Lee, Min Ju Lee, Hans Jin-young Oh, Ah Jung Heo, Do Hyun Han, Yong Tae Kwon, and Chang Hoon Ji

Document S1

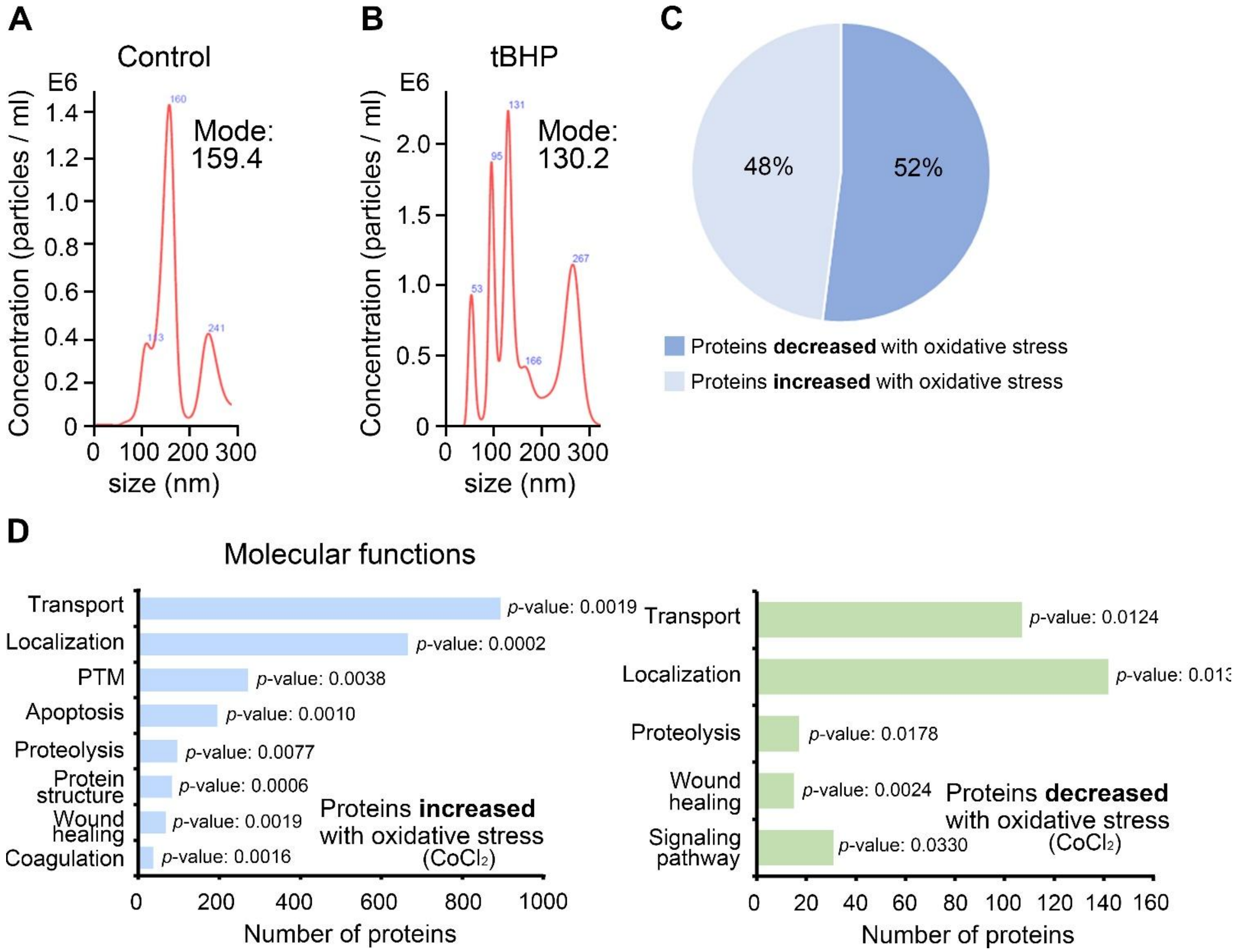

Figure S1. The molecular functions of oxidative stress-induced exosomes.

(A-B) Nanoparticle tracking analysis of exosomes extracted from A549 cells in the presence or absence of tBHP (250 μM, 6 h).

(C) A pie chart illustrating the distribution of proteins showing increased, decreased, and no significant changes in response to oxidative stress.

(D) Bar graph of Gene Ontology (GO) enrichment analysis. Enrichment of gene ontology terms within molecular pathways in increased and decreased exosomal proteins with $CoCl_2$ (250 μM, 6 h). *p*-values are indicated above the graph.

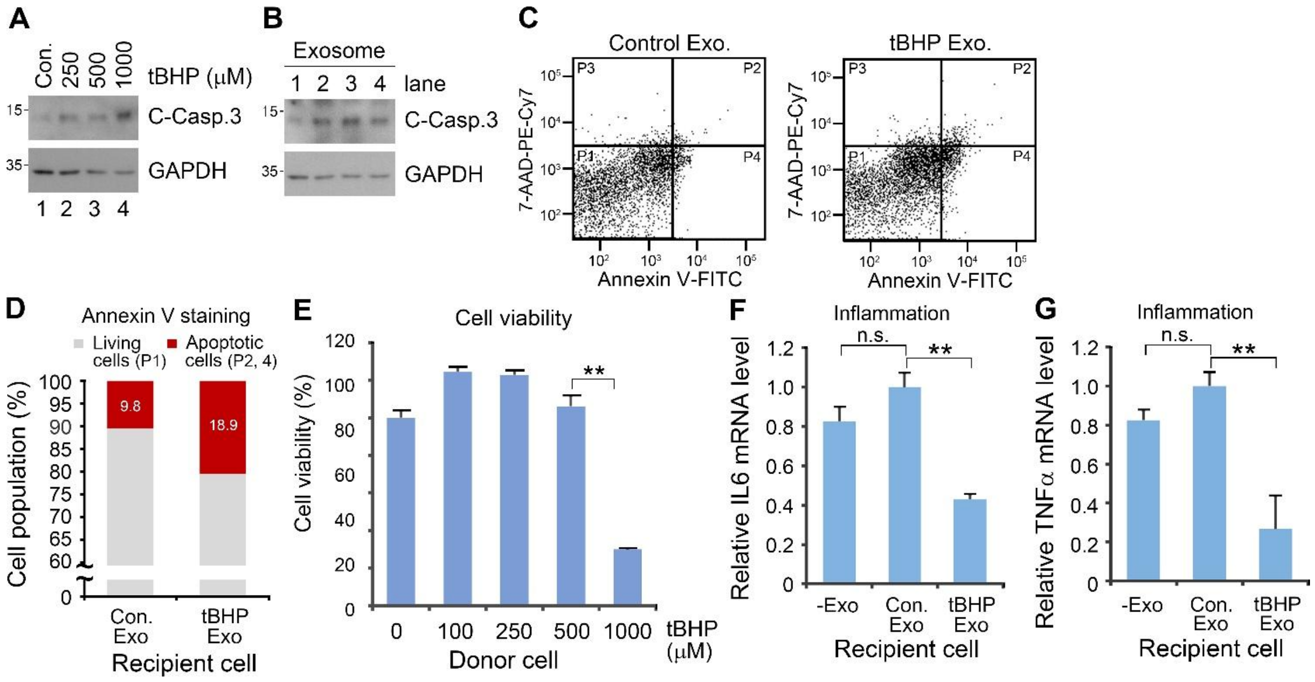

Figure S2. Oxidative stress-induced exosomes are pro-apoptotic and anti-inflammatory.

(A) Immunoblotting analysis of A549 cells with 250, 500, and 1000 μM of tBHP treatment (6 h).

(B) Immunoblotting analysis of A549 cells treated with exosomes from **A** (24 h).

(C) Annexin V/7-AAD flow cytometry assay of A549 cells treated with exosomes from A549 cells in the presence or absence of tBHP (250, 500 μM, 6 h) treatment.

(D) Quantification of **C** (n > 6000). P1 was defined as living cells and the sum of P2 and P4 was defined as apoptotic cells.

(E) Cellular viability of recipient A549 cells treated with various concentrations of tBHP (100, 250, 500, and 1000 μM, 6 h). Results are presented as OD values (absorbance at 450 nm).

(F) Relative mRNA level of *IL-6* in A549 cells treated with exosomes originated from donor cells in the presence or absence of tBHP (250 μM, 6 h) treatment compared with control.

(G) Relative mRNA level of *TNF*α in A549 cells treated with exosomes originated from donor cells in the presence or absence of tBHP (250 μM, 6 h) treatment compared with control. Error bars represent SEM (n=3 replicates). **p<0.01 using the paired t-test.

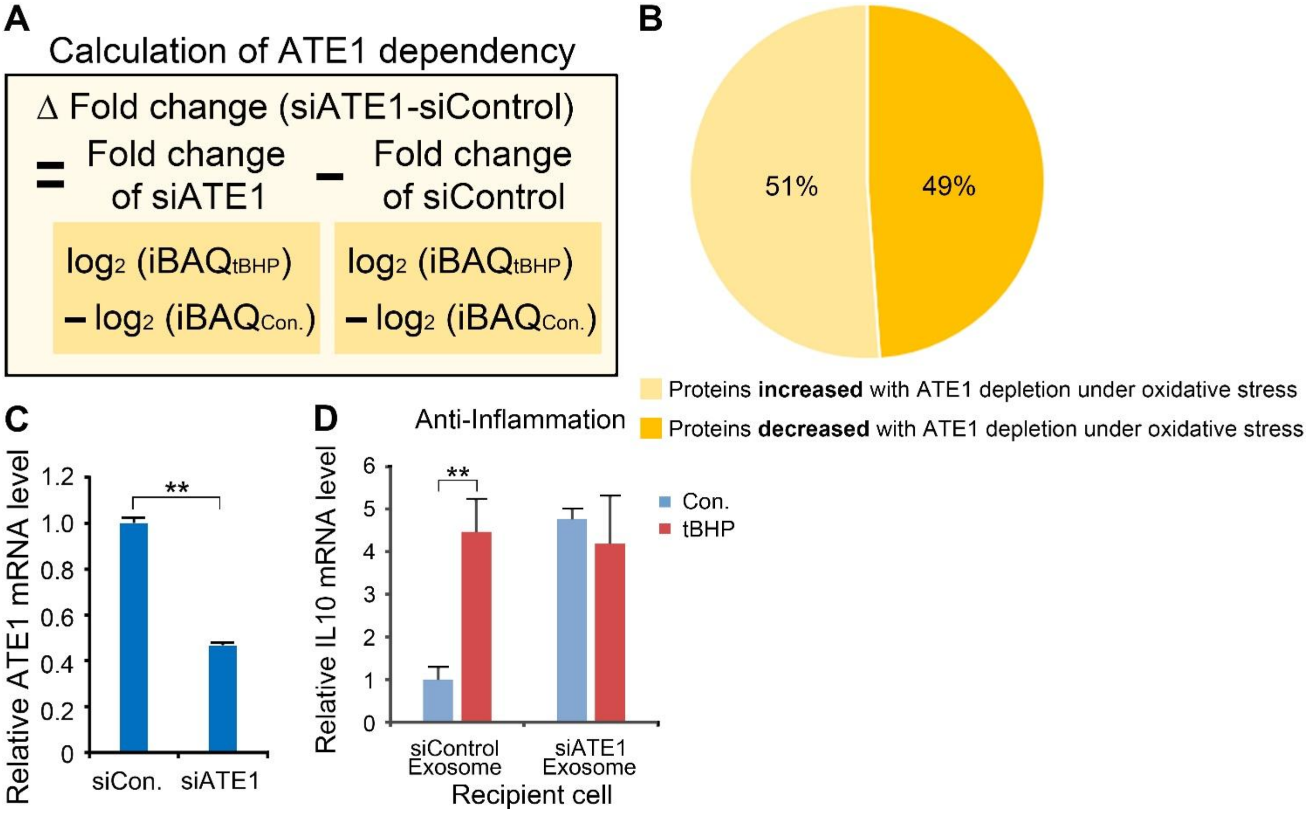

Figure S3. ATE1 dependency of exosomal proteomes under oxidative stress.

(A) Intensity-based absolute quantification (iBAQ) formula to calculate ATE1 dependency of exosomal targeting for each cargo.

(B) Illustration of proteinsthe protein distribution showing increased, decreased in response to oxidative stress.

(C) Relative mRNA level of *ATE1* in A549 cells with genetic inhibition of *ATE1* compared with control. Error bars represent SEM (n=3 replicates).

(D) Relative mRNA level of *IL-10* in A549 cells treated with exosomes originated from A549 cells in RNA interference of *ATE1* with tBHP (500 μM, 6 h) treatment. **p<0.01 using the paired t-test.

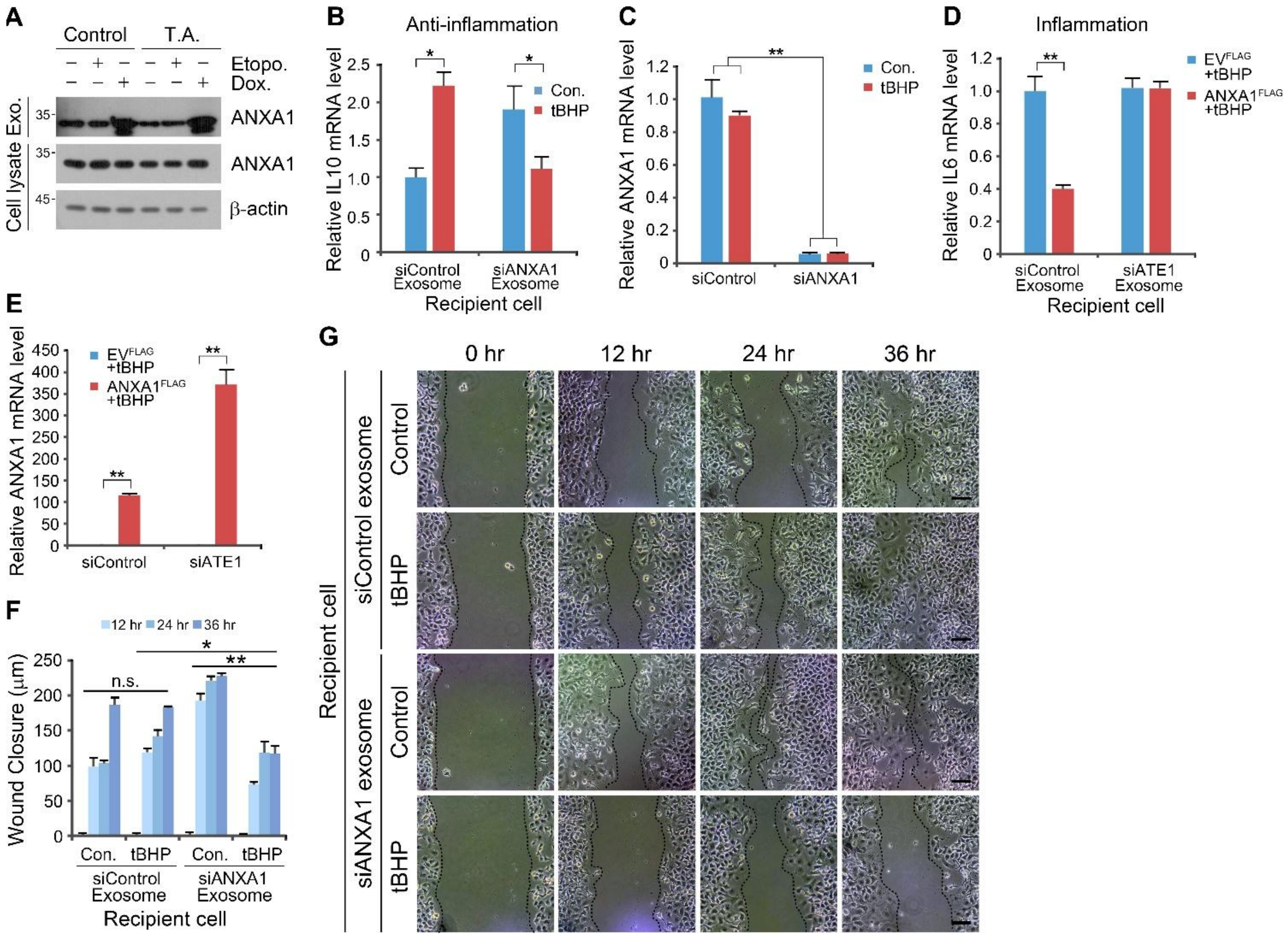

Figure S4. ATE1-ANXA1 circuit mediates anti-inflammation and pro-proliferation by cancer cell-derived exosomes under oxidative stress.

(A) Immunoblotting analysis of cell lysate and exosome fraction ANXA1 and β-actin in A549 cells with Etoposide (2 μM, 24 h), Doxorubicin (2 μM, 24 h), and Tannic acid (30 μM, 24 h) treatment.

(B) Relative mRNA level of *IL-10* in A549 cells treated with exosomes originated from A549 cells in RNA interference of *ANXA1* with tBHP (250 μM, 6 h) treatment.

(C) Relative mRNA level of *ATE1* in A549 cells with genetic inhibition of *ATE1* compared with control.

(D) Relative mRNA level of *IL-6* in A549 cells treated with exosomes originated from cells under *ATE1* knockdown and ANXA1^{-FLAG} overexpression with tBHP (250 μM, 6 h) treatment.

(E) Relative mRNA level of *ANXA1* in A549 cells under *ATE1* knockdown and ANXA1-FLAG overexpression with tBHP (250 μM, 6 h) treatment.

(F) Bar graphs of wound closure evaluated by measuring the remaining cell-free area at 12, 24, and 36 h after the wound.

(G) Representative light microscope images of Wound healing assays for A549 to evaluate migration rate at 12, 24, and 36 h, after treatment of exosomes extracted from A549 cells under *ANXA1* knockdown and tBHP (250 mM, 6 h). Original magnification 20×, scale bar 50 mm.

Error bars represent SEM (n=3 replicates). *p<0.05, **p<0.01 using the paired t-test.

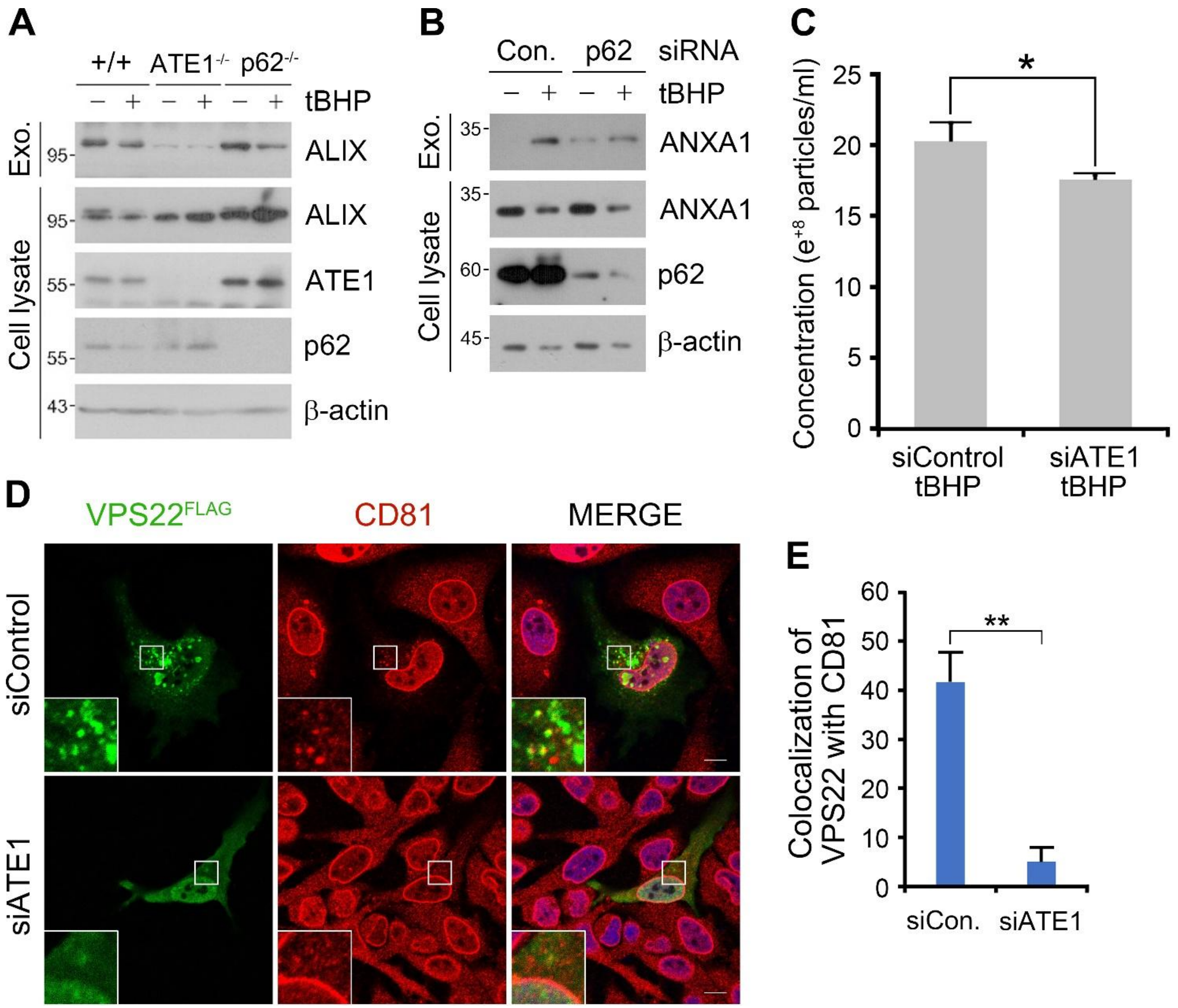

Figure S5. ATE1-mediated RILP cleavage recruits ESCRT-II machinery for exosome biogenesis under oxidative stress.

(A) Immunoblotting analysis of cell lysate and exosome fraction ALIX, ATE1, p62, and β-actin in +/+, *ATE1*$^{-/-}$, *p62*$^{-/-}$ HeLa cells with tBHP treatment (6 h).

(B) Immunoblotting analysis of cell lysate and exosome fraction ANXA1, p62, and β-actin in A549 cells under *p62* knockdown and tBHP treatment (250 μM, 6 h).

(C) The concentration of exosome particles measured by nano-trafficking analysis. Exosomes were isolated from A549 cells in the presence or absence of RNA-mediated interference of *ATE1* with tBHP treatment (250 μM, 6 h).

(D) Co-localization immunostaining analysis of VPS22 with CD81 in HeLa cells transfected with VPS22^{-FLAG} under *ATE1* knockdown. Scale bar, 10 μm.

(E) Quantification of **D**.

Error bars represent SEM (n=50 cells). **$p<0.01$ using the paired t-test.

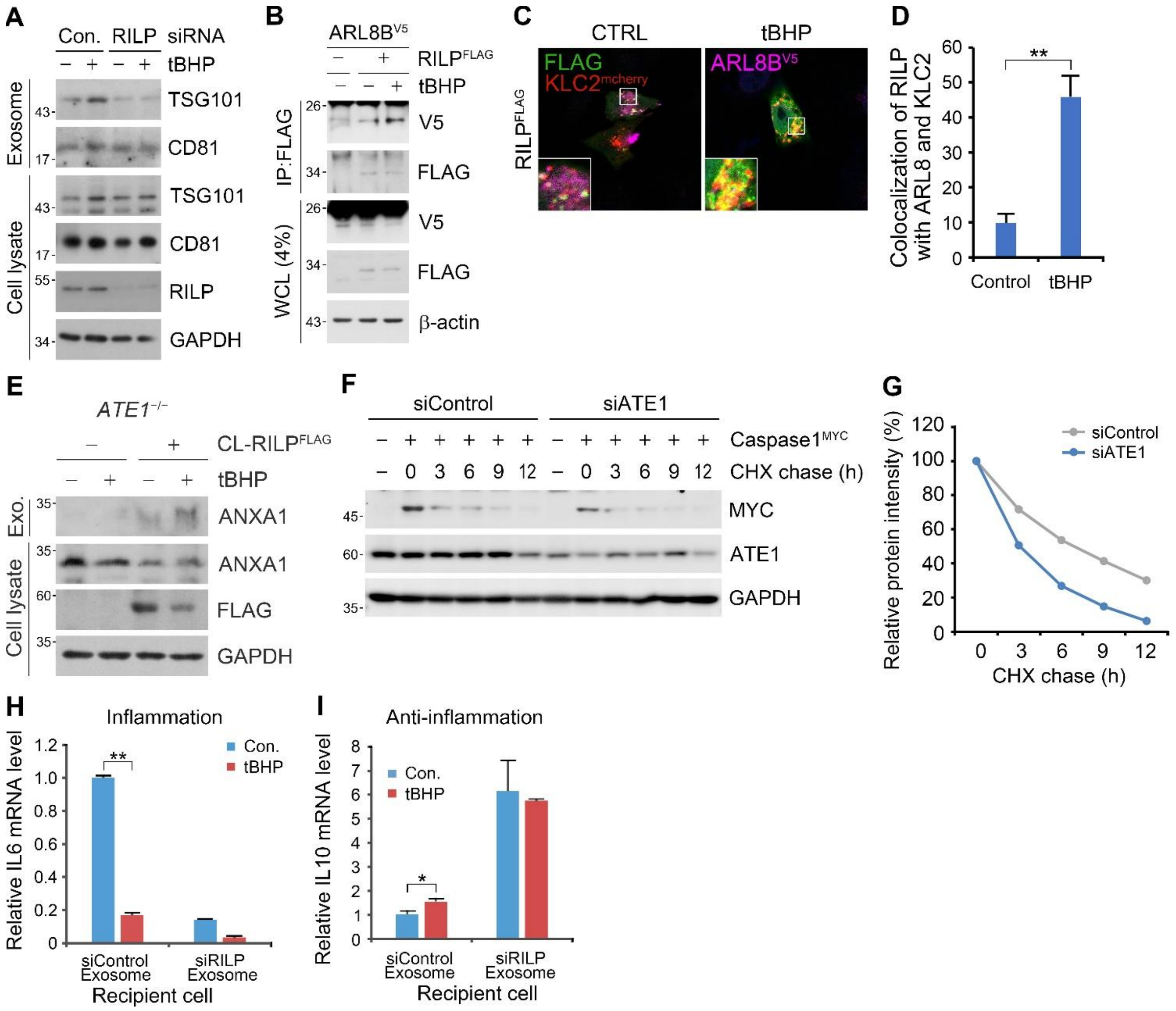

Figure S6. The Arg/N-degron pathway mediates caspase-1 stabilization and RILP cleavage.

(A) Co-immunoprecipitation (Co-IP) analysis of the interaction between RILP^{-FLAG} and ARL8B^{-V5} in HEK293T cells followed by tBHP (250 μM, 6 h) treatment.

(B) Immunoblotting analysis of cell lysate and exosome fraction TSG101, CD81, RILP, and GAPDH in A549 cells with RNA interference of *RILP* and 250 μM tBHP treatment (6 h).

(C) Co-localization immunostaining analysis of RILP^{-FLAG} with ARL8B^{-V5} and KLC2$^{-mcherry}$ in HeLa cells in the presence or absence of tBHP treatment (250 μM, 6 h). Scale bar, 10 μm.

(D) Quantification of **C** (n=50).

(E) Immunoblotting analysis of cell lysate and exosome fraction ANXA1, FLAG, and GAPDH in *ATE1*$^{-/-}$ HeLa cells with Cleaved (CL)-RILP^{-FLAG} transfection and tBHP treatment (250 μM, 6 h).

(F) Cycloheximide chase assay in A549 cells with transient expression of caspase-1^{-MYC} and RNAi interference of *ATE1*.

(G) Quantification of **F**.

(H) Relative mRNA level of *IL-6* in A549 cells treated with exosomes originated from A549 cells in RNA interference of *RILP* with tBHP (500 μM, 6 h) treatment.

(I) Relative mRNA level of *IL-10* in A549 cells treated with exosomes originated from A549 cells in RNA interference of *RILP* with tBHP (500 μM, 6 h) treatment. Error bars represent SEM (n=50 cells). **p<0.01 using the paired t-test.

Table S1. List of proteins identified by mass analysis.

LC-MS/MS analysis of exosomes isolated from cells subjected to RNA interference of *ATE1* and treated with tBHP (250 μM, 6 h), compared to control cells. Fold changes and *P*-values were calculated by comparing control cells with tBHP-treated cells. *ATE1* dependency was determined using the method described in Figure S3A.

[The table is uploaded as an Excel file.]

Table S2. Gene Ontology (GO) analysis of biological processes associated with proteins identified in exosomes.

LC-MS/MS analysis of exosomes isolated from cells with treatment of tBHP (250 μM, 6 h) or $CoCl_2$ (250 μM, 24 h), compared to control cells.

Increased upon tBHP				Increased upon tBHP			
Molecular function	Count	p-value	-log (p-value)	Molecular function	Count	p-value	-log (p-value)

inflammation	42	0.0335	1.4750	wound healing	11	0.0017	2.7787
apoptosis	36	0.0495	1.3056	proteolysis	7	0.0127	1.8977
endocytosis	13	0.0327	1.4852	protein complex	16	0.0403	1.3951
wound healing	10	0.0356	1.4489	proliferation	24	0.0345	1.4625
coagulation	8	0.0325	1.4878	signaling pathway	8	0.0438	1.3585
Increased upon $CoCl_2$				Increased upon $CoCl_2$			
Molecular function	Count	p-value	-log (p-value)	Molecular function	Count	p-value	-log (p-value)
transport	894	0.0019	2.7173	transport	107	0.0124	1.9060
localization	665	0.0002	3.7888	localization	142	0.0134	1.8743
PTM	271	0.0038	2.4243	proteolysis	17	0.0178	1.7488
apoptosis	195	0.0010	2.9949	wound healing	15	0.0024	2.6195
proteolysis	96	0.0077	2.1113	signaling pathway	31	0.0330	1.4818
protein structure	83	0.0006	3.2447				
wound healing	67	0.0019	2.7191				
coagulation	37	0.0016	2.7950				
